# Oral ixazomib-dexamethasone vs oral pomalidomide-dexamethasone for lenalidomide-refractory, proteasome inhibitor-exposed multiple myeloma: a randomized Phase﻿ 2 trial

**DOI:** 10.1038/s41408-021-00593-2

**Published:** 2022-01-24

**Authors:** Meletios A. Dimopoulos, Fredrik Schjesvold, Vadim Doronin, Olga Vinogradova, Hang Quach, Xavier Leleu, Yolanda Gonzalez Montes, Karthik Ramasamy, Alessandra Pompa, Mark-David Levin, Cindy Lee, Ulf Henrik Mellqvist, Roland Fenk, Hélène Demarquette, Hamdi Sati, Alexander Vorog, Richard Labotka, Jichang Du, Mohamed Darif, Shaji Kumar

**Affiliations:** 1 Hematology & Medical Oncology, Department of Clinical Therapeutics, National and Kapodistrian University of Athens, School of Medicine, Athens, Greece; 2 Oslo Myeloma Center, Oslo University Hospital, Oslo, Norway; 3 KG Jebsen Center for B Cell Malignancies, University of Oslo, Oslo, Norway; 4 Department of Hematology, City Clinical Hospital No. 40, Moscow, Russia; 5 SBIHM “City Clinical Hospital n.a. S. P. Botkin of DHM”, City Hematologic Center, Moscow, Russia; 6 Department of Haematology, University of Melbourne, St. Vincent’s Hospital, Melbourne, VIC Australia; 7 Pôle Régional de Cancérologie, CHU and Inserm, 1402 Poitiers, France; 8 Department of Haematology, Josep Trueta ICO Girona Hospital, Girona, Spain; 9 Department of Haematology, Oxford University Hospitals NHS Trust, Oxford, United Kingdom; 10 Department of Haematology, GenesisCare, Oxford, United Kingdom; 11 Division of Hematology and Stem Cell Transplantation, Fondazione Ca’Granda IRCCS Ospedale Maggiore Policlinico, Milan, Italy; 12 Department of Internal Medicine, Albert Schweitzer Hospital, Dordrecht, Netherlands; 13 Department of Haematology, The Queen Elizabeth Hospital, Adelaide, SA Australia; 14 Department of Internal Medicine, South Elvsborg Hospital, Boras, Sweden; 15 Department of Hematology, Oncology and Clinical Immunology, University Hospital Duesseldorf, Dusseldorf, Germany; 16 Centre Hospitaliser de Dunkerque, Dunkerque, France; 17 Department of Haematology, Singleton Hospital, Swansea Bay University Health Board, Swansea, United Kingdom; 18 Takeda Development Center Americas, Inc. (TDCA), Lexington, MA USA; 19 Division of Hematology, Department of Internal Medicine, Mayo Clinic, Rochester, MN USA

**Keywords:** Disease-free survival, Myeloma, Adverse effects, Combination drug therapy, Phase II trials

## Abstract

Multiple myeloma (MM) patients typically receive several lines of combination therapy and first-line treatment commonly includes lenalidomide. As patients age, they become less tolerant to treatment, requiring convenient/tolerable/lenalidomide-free options. Carfilzomib and/or bortezomib-exposed/intolerant, lenalidomide-refractory MM patients with ≥2 prior lines of therapy were randomized 3:2 to ixazomib-dexamethasone (ixa-dex) (*n* = 73) or pomalidomide-dexamethasone (pom-dex) (*n* = 49) until progression/toxicity. Median progression-free survival (mPFS) was 7.1 vs 4.8 months with ixa-dex vs pom-dex (HR 0.847, 95% CI 0.535–1.341, *P* = 0.477; median follow-up: 15.3 vs 17.3 months); there was no statistically significant difference between arms. In patients with 2 and ≥3 prior lines of therapy, respectively, mPFS was 11.0 vs 5.7 months (HR 1.083, 95% CI 0.547–2.144) and 5.7 vs 3.7 months (HR 0.686, 95% CI 0.368–1.279). Among ixa-dex vs pom-dex patients, 69% vs 81% had Grade ≥3 treatment-emergent adverse events (TEAEs), 51% vs 53% had serious TEAEs, 39% vs 36% had TEAEs leading to drug discontinuation, 44% vs 32% had TEAEs leading to dose reduction, and 13% vs 13% died on study. Quality of life was similar between arms and maintained during treatment. Ixa-dex represents an important lenalidomide-free, oral option for this heavily pretreated, lenalidomide-refractory, proteasome inhibitor-exposed population.

**Trial registration:** ClinicalTrials.gov number, NCT03170882.

## Introduction

For most patients with multiple myeloma (MM), the development of relapsed and refractory MM (RRMM) is inevitable, requiring several lines of therapy with multiple drug combinations throughout the course of treatment [1–3]. Consequently, the disease may become refractory to more than one agent, and patients receiving their third or later line of therapy are likely to have poorer responses than in earlier lines, making treatment more challenging [4–10].

RRMM is a heterogeneous disease, and no single treatment regimen is effective for all patients [1, 2, 11]. Since lenalidomide-containing regimens are common as first-line therapy in MM [1, 2, 12], lenalidomide-free options for subsequent lines are needed as patients become refractory to this agent [13–15]. Several lenalidomide-free triplet regimens are approved in this setting [14–23]. These combinations, however, are parenterally administered, increasing the treatment burden for the already heavily pretreated patient, suggesting a need for options that can limit hospital or clinic visits [24]. Residual comorbidities or impairments from previous therapies are common in this population [25], and, as patients have aged several years since therapy onset, they are likely to be frailer and potentially less able to tolerate treatment toxicity [10, 12, 25], notably in the context of standard-of-care triplet regimens [19, 26–29]. Therefore, there is a need for more convenient regimens, such as all-oral doublets, that are active, have manageable toxicity, and do not adversely impact quality of life (QoL).

Dexamethasone-based doublets have been shown to be effective and tolerable in patients with RRMM [30, 31], and pomalidomide-dexamethasone (pom-dex) is indicated for use in adults with RRMM who have received ≥2 prior treatment regimens, including both lenalidomide and bortezomib, and have progressed on their last therapy [30]. However, treatment with pom-dex is associated with Grade ≥3 hematological toxicities and infections [18, 32]. In addition, for a patient population previously exposed to pom-dex-based triplets or those who have become refractory to pomalidomide, alternative and effective all-oral doublet options may be required.

Ixazomib, the first oral proteasome inhibitor (PI), is approved in the United States and European Union in combination with lenalidomide-dexamethasone for MM patients who have received ≥1 prior line of therapy [33, 34]. Ixazomib is well tolerated as long-term therapy, with predictable and manageable toxicities [26]. A Phase 2 study in which RRMM patients with limited or no prior exposure to bortezomib were treated with ixazomib 4 or 5.5 mg weekly for 3 of 4 weeks in combination with dexamethasone 40 mg weekly reported overall response rates (ORRs) of 31% for the ixazomib 4 mg arm and 54% for the 5.5 mg arm, with both arms demonstrating manageable toxicities. In that study, the authors suggest that ixazomib-dexamethasone (ixa-dex), at either dose of ixazomib, may provide greater clinical benefit compared with pom-dex in this population of patients with limited prior exposure to a PI [35, 36]. These findings provided the rationale for the present global, multicenter, open-label, randomized, Phase 2 study of ixa-dex vs pom-dex in adult patients with lenalidomide-refractory, PI-exposed MM.

## Methods

### Patients

Eligible patients had a confirmed diagnosis of MM per International Myeloma Working Group (IMWG) criteria, an Eastern Cooperative Oncology Group performance score of 0–2, had relapsed or progressed after ≥2 prior lines of therapy, and were lenalidomide-refractory. Patients had to have achieved ≥partial response (PR) to carfilzomib or bortezomib, or had to have discontinued treatment with either PI due to intolerance (see Supplementary Table 1 for detailed eligibility criteria).

### Study design

In this international, multicenter, open-label Phase 2 study, patients were centrally randomized (3:2) via interactive response technology to receive either ixa-dex or pom-dex in 28-day cycles. Patients on the ixa-dex arm received oral ixazomib 4 mg on Days 1, 8, and 15, and oral dexamethasone 20 mg (10 mg in patients aged ≥75 years at randomization) on Days 1, 2, 8, 9, 15, 16, 22, and 23. The starting dose of ixazomib 4 mg was selected based on the dose used in TOURMALINE-MM1 [26], with escalation to 5.5 mg at Cycle 2 if the 4 mg dose was well tolerated [35, 36]. Patients on pom-dex received oral pomalidomide 4 mg on Days 1–21 and oral dexamethasone 40 mg (20 mg in patients aged ≥75 years at randomization) on Days 1, 8, 15, and 22 as per license. Patients continued treatment until progressive disease (PD) or unacceptable toxicity. Randomization was stratified by age (<65 vs ≥65 years), International Staging System (ISS) disease stage at study entry (I or II vs III), and number of prior lines of therapy (2 vs ≥3).

The primary endpoint was progression-free survival (PFS), defined as time from randomization to first documentation of PD as assessed by the investigator per IMWG criteria [37], or death from any cause, whichever occurred first. Secondary endpoints were overall survival (OS), ORR (≥ PR), duration of response (DOR), time to response (TTR), time to progression (TTP), health-related QoL (HRQoL), healthcare resource utilization (HRU), and safety.

The trial was conducted in accordance with the Declaration of Helsinki, International Conference on Harmonization Good Clinical Practice guideline, and appropriate regulatory requirements. Local ethics committees or institutional review boards approved the protocol and all patients provided written informed consent.

### Assessments

Response assessments were performed every cycle until PD or every 4 weeks in patients who discontinued treatment prior to PD. Response and disease progression assessments were based on central laboratory results and IMWG 2011 criteria [38]. A single bone marrow (BM) aspirate or biopsy disease assessment was performed locally at screening and repeated if the patient showed evidence of complete response (CR), or to investigate suspected PD. To confirm suspected CR, BM immunohistochemistry or immunofluorescence for kappa/lambda ratio was performed. Following PD, patients were followed up every 12 weeks for subsequent therapy and OS.

Safety was assessed throughout the study, and treatment-emergent adverse events (TEAEs) were graded using the National Cancer Institute’s Common Terminology Criteria for Adverse Events, version 4.03. If peripheral neuropathy (PN) occurred, each subsequent monthly evaluation recorded the grade of PN at that visit. PN was followed monthly until either resolution, initiation of a subsequent line of therapy, or 6 months after PD, whichever occurred first.

HRQoL was evaluated through patient self-reported instruments including the European Organization for Research and Treatment of Cancer (EORTC) Quality of Life Questionnaire (QLQ) Core-30 (C30) and myeloma-specific (MY-20) instruments, and the 5-level classification system of the EuroQol 5-Dimensional Health Questionnaire (EQ-5D-5L). These assessments were obtained at screening and at every cycle until PD. In patients who discontinued treatment prior to PD, assessments were made every 4 weeks, and the EQ-5D-5L was completed every 12 weeks. HRU data included medical visits (number and rates, reasons for, and length of stay): hospitalizations, emergency room visits, and outpatient visits.

### Statistical analysis

The analyses of the primary endpoint, PFS, and the secondary endpoints of TTP and OS were conducted in the intent-to-treat (ITT; all randomized patients) population. Kaplan–Meier methodology was used to estimate PFS, and OS distributions in each treatment arm, as well as TTP distributions. DOR was summarized descriptively for responders using the Kaplan–Meier method; TTR was compared in the ITT population and summarized descriptively for the responders. Patients without documentation of PD were censored at the date of the last response assessment (≥stable disease on PFS, TTP and DOR analyses), and patients without documentation of death at the time of OS analysis were censored at the date last known to be alive. Kaplan–Meier medians, plus 95% confidence intervals (CIs), were determined, if estimable. An unadjusted, unstratified Cox model was used to estimate the hazard ratio (HR) and 95% CI for the treatment effect, and a 2-sided, unstratified log-rank test was used to compare treatment groups for each endpoint. Subgroup analyses were conducted for PFS and OS relative to baseline stratification factors, demographic data, and disease characteristics.

A sample size of ~120 patients was determined in order to give ~81 events for the PFS analysis, which provided 80% power at a 2-sided alpha of 0.20 to detect a difference between arms; this was based on an assumption of a HR of 0.62 and a median PFS of 7.3 months for ixa-dex vs 4.5 months for pom-dex (estimated based on previous published data in similar populations [36, 39]). OS was to be tested at 2-sided alpha of 0.20; the study was not powered for OS comparisons.

ORR was defined as the proportion of patients who achieved ≥PR in the ITT population. A logistic regression model was used to estimate the treatment effect in terms of odds ratio (OR) and its 95% CIs.

This report represents the final analysis of the study. A Phase 3 portion in a larger sample size was planned, but this was removed following a protocol amendment in response to slower-than-projected accumulation of PFS events and contemporaneous advances in standard-of-care approaches in the RRMM treatment paradigm.

## Results

### Patients

**Fig. 1:**
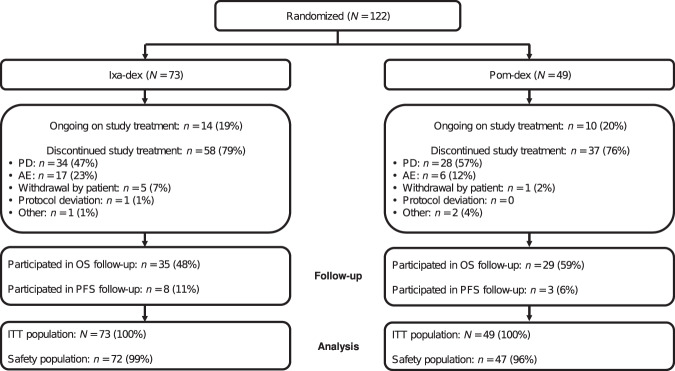
CONSORT diagram. Patient disposition throughout the study. AE adverse event, dex dexamethasone, ITT intent-to-treat, ixa ixazomib, OS overall survival, PD progressive disease, PFS progression-free survival, pom pomalidomide.

Between February 28, 2018, and October 3, 2019, 122 patients from 51 sites in 13 countries in Europe and the Middle East, and 3 sites in Australia were randomized: 73 to the ixa-dex arm and 49 to the pom-dex arm (Fig. 1). Baseline demographics and disease characteristics were generally well-balanced between the two treatment arms (Table 1). Median age in patients treated with ixa-dex vs pom-dex was 72 vs 68 years (75% vs 73% were aged ≥65 years and 36% vs 18% were aged ≥75 years), 25% vs 22% of patients had ISS stage III MM, and 52% vs 53% had received ≥3 prior lines of therapy per stratification (prior lines of therapy are detailed in Supplementary Table 2).

**Table 1:** Patient demographics and baseline disease characteristics.

	Ixa-dex *N* = 73	Pom-dex *N* = 49
Median age (range), years	72 (42–86)	68 (39–83)
Age categories, *n* (%)		
<65 years^a^	18 (25)	13 (27)
≥65 years^a^	55 (75)	36 (73)
≥65–<75 years	29 (40)	27 (55)
≥75–<85 years	23 (32)	9 (18)
≥85 years	3 (4)	0
Sex, *n* (%)		
Male	35 (48)	26 (53)
Female	38 (52)	23 (47)
Race, *n* (%)		
White	69 (95)	46 (94)
Asian	1 (1)	0
Black or African American	1 (1)	0
Not reported	2 (3)	3 (6)
ECOG performance status at baseline, *n* (%)		
0	28 (38)	21 (43)
1	38 (52)	23 (47)
2	7 (10)	5 (10)
ISS stage at study entry,^a^ *n* (%)		
I or II	55 (75)	38 (78)
III	18 (25)	11 (22)
Creatinine clearance, *n* (%)		
≥30–<60 mL/min	21 (29)	12 (24)
≥60 mL/min	27 (37)	15 (31)
≥90 mL/min	19 (26)	10 (20)
Missing	6 (8)	12 (24)
LDH status, *n* (%)		
Elevated	13 (18)	9 (18)
Normal	59 (81)	40 (82)
Missing	1 (1)	0
Median time from initial diagnosis to first dose of study treatment, months (range)	67.2 (22–169)	58.6 (6–274)
Number of prior lines of therapy,^a^ *n* (%)		
2	35 (48)	23 (47)
≥3	38 (52)	26 (53)
Relapsed and/or refractory status to prior systemic therapy, *n* (%)		
Relapsed	2 (3)	2 (4)
Refractory	16 (22)	11 (22)
Relapsed and refractory	55 (75)	36 (73)
Prior lines of therapy received, *n* (%)		
Bortezomib	73 (100)	49 (100)
Lenalidomide	73 (100)	49 (100)
Thalidomide	23 (32)	10 (20)
Daratumumab	14 (19)	7 (14)
Carfilzomib	7 (10)	1 (2)
Prior transplant, *n* (%)	32 (44)	28 (57)

*Dex*, dexamethasone, *ECOG* Eastern Cooperative Oncology Group, *ISS* International Staging System, *ixa* ixazomib, *LDH* lactate dehydrogenase, *pom* pomalidomide.

^a^Stratification factor.

At data cutoff (May 31, 2020), 19% ixa-dex vs 20% pom-dex patients were ongoing on study treatment (Fig. 1). Study treatment had been discontinued in 79% vs 76% patients; primary reasons for discontinuation were PD in 47% vs 57%, and adverse events (AEs) in 23% vs 12% of patients (Fig. 1).

### Efficacy

**Fig. 2:**
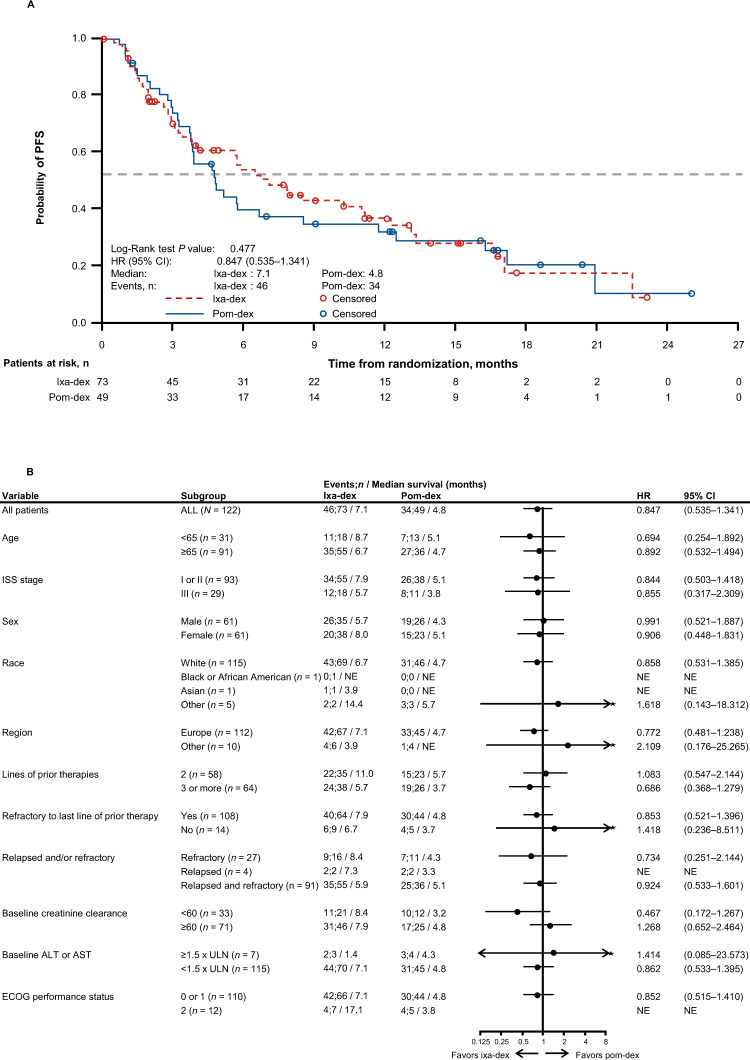
PFS with ixa-dex vs pom-dex in the ITT population. **A** Kaplan–Meier analysis of PFS and (**B**) Forest plot of PFS in prespecified subgroups based on patient and disease characteristics. ALT alanine aminotransferase, AST aspartate aminotransferase, CI confidence interval, dex dexamethasone, ECOG Eastern Cooperative Oncology Group, HR hazard ratio, ISS International Staging System, ITT intent-to-treat, ixa ixazomib, NE not estimable, PFS progression-free survival, pom pomalidomide, ULN upper limit of normal.

With 46 (63%) and 34 (69%) PFS events in the ixa-dex and pom-dex arms, respectively, median PFS was 7.1 months (95% CI 3.9–11.1) vs 4.8 months (95% CI 3.7–8.5), and the HR was 0.847 (95% CI 0.535–1.341; *P* = 0.477; Fig. 2A). Median PFS with ixa-dex vs pom-dex in patients with 2 prior lines of therapy was 11.0 vs 5.7 months (HR 1.083, 95% CI 0.547–2.144); in patients with ≥3 prior lines, median PFS was 5.7 vs 3.7 months (HR 0.686, 95% CI 0.368–1.279). Figure 2B shows PFS with ixa-dex vs pom-dex in all prespecified subgroups based on patient and disease characteristics.

Although age (<65 vs ≥65 years) was a stratification factor, an imbalance in the number of elderly patients was observed in the ixa-dex vs pom-dex arms (32% vs 18% of patients were aged ≥75 to ≤84 years, and 4% vs 0% were aged ≥85 years). Consequently, an ad-hoc analysis of PFS with ixa-dex vs pom-dex in patients aged <75 years (median 6.5 vs 4.8 months, HR 0.843, 95% CI 0.491–1.448) and ≥75 years (median 8.0 vs 4.7 months, HR 0.890, 95% CI 0.309–2.562) is provided in Supplementary Fig. 1.

Overall, 28 vs 20 patients had ≥PR to treatment with ixa-dex vs pom-dex for an ORR of 38% vs 41% (OR 0.90, 95% CI 0.43–1.90) with ≥very good PR (VGPR) rates of 7% vs 16% (Table 2). Among responding patients, median TTR was 2.0 vs 1.1 months (HR 0.556, 95% CI 0.288–1.073) and median DOR was 14.8 vs 14.3 months (Supplementary Fig. 2).

**Table 2:** Best confirmed responses to ixa-dex and pom-dex in the ITT population.

Confirmed best response	Ixa-dex *N* = 73 *n* (%) [Exact 95% CI]	Pom-dex *N* = 49 *n* (%) [Exact 95% CI]	OR [95% CI]	*P* value
ORR (CR + PR + VGPR)	28 (38) [27–50]	20 (41) [27–56]	0.90 [0.43–1.90]	0.634
CR + VGPR	5 (7) [2–15]	8 (16) [7–30]	0.36 [0.11–1.20]	0.080
CR	0	2 (4) [<1–14]	NE	0.058
sCR	0	0		
VGPR	5 (7) [2–15]	6 (12) [5–25]		
PR	23 (32) [21–43]	12 (24) [13–39]		
SD	33 (45) [34–57]	19 (39) [25–54]		
PD	8 (11) [5–20]	5 (10) [3–22]		

*CI* confidence interval, *CR* complete response, *dex* dexamethasone, *ITT* intent-to-treat, *ixa* ixazomib, *NE* not estimable, *OR* odds ratio, *ORR* overall response rate, *PD* progressive disease, *pom* pomalidomide, *PR* partial response, *sCR* stringent complete response, *SD* stable disease, *VGPR* very good partial response.

**Fig. 3:**
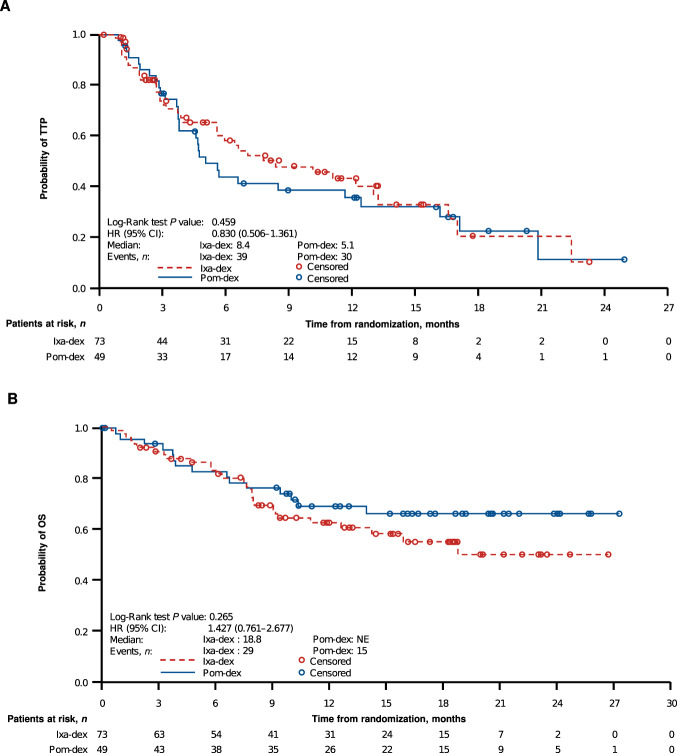
Kaplan–Meier analysis of TTP and OS with ixa-dex vs pom-dex in the ITT population. **A** TTP and (**B**) OS. CI confidence interval, dex dexamethasone, HR hazard ratio, ITT intent-to-treat, ixa ixazomib, NE not estimable, OS overall survival, pom pomalidomide, TTP time to progression.

Median TTP was 8.4 vs 5.1 months (HR 0.830, 95% CI 0.506–1.361) with ixa-dex vs pom-dex (Fig. 3A). After a median follow-up of 15.3 and 17.3 months, and with 29 (40%) and 15 (31%) patients having died in the ixa-dex and pom-dex arms, respectively, median OS was 18.8 months vs not reached (HR 1.427, 95% CI 0.761–2.677; Fig. 3B). OS in patients aged <75 and ≥75 years is shown in Supplementary Fig. 1, and the forest plot of OS in prespecified patient subgroups is shown in Supplementary Fig. 3.

Overall, 30 (42%) vs 23 (49%) of patients in the ixa-dex vs pom-dex arms had started subsequent lines of therapy at data cutoff for this analysis (Supplementary Table 3). Subsequent therapy containing immunomodulatory drugs was received by 32% vs 9% of patients, including 28% vs 4% who received pom; by contrast, 10% vs 36% of patients received PIs, and 19% vs 28% received monoclonal antibodies.

### Treatment exposure and safety

The safety population included 72 and 47 patients in the ixa-dex and pom-dex arms, respectively (Fig. 1). Patients received a median of 6 cycles in both the ixa-dex (range 1–25) and pom-dex (range 1–27) arms, with 7% and 15%, respectively, completing ≥19 cycles (Table 3). Mean relative dose intensities were numerically lower with ixa-dex vs pom-dex. Overall, 64% of patients receiving ixa-dex escalated to a 5.5 mg dose of ixazomib (Table 3).

**Table 3:** Treatment exposure and overall safety profile with ixa-dex and pom-dex in the safety population.

	Ixa-dex *n* = 72	Pom-dex *n* = 47
Treatment exposure		
Median number of treatment cycles received, *n* (range)	6 (1–25)	6 (1–27)
Median time on treatment at ixa 5.5 mg, months (range)	*n﻿* = 46 1.8 (<1–17)	
Patients receiving ≥7 cycles, *n* (%)	35 (49)	21 (45)
Patients receiving ≥19 cycles, *n* (%)	5 (7)	7 (15)
Mean (StD) relative dose intensity, %		
Ixazomib / Pomalidomide	79.5 (15.02)	89.9 (13.31)
Dexamethasone	84.0 (16.62)	87.8 (15.57)
Safety profile		
Any TEAE, n (%)	70 (97)	47 (100)
Any drug-related TEAE, *n* (%)	64 (89)	39 (83)
Any Grade ≥3 TEAE, *n* (%)	50 (69)	38 (81)
Any drug-related Grade ≥3 TEAE, *n* (%)	35 (49)	31 (66)
Any serious TEAE, *n* (%)	37 (51)	25 (53)
Any drug-related serious TEAE, *n* (%)	16 (22)	13 (28)
TEAE resulting in dose reduction of ≥1 of the 2 agents in the study drug regimen, *n* (%)	32 (44)	15 (32)
TEAE resulting in discontinuation of ≥1 of the 2 agents in the study drug regimen, *n* (%)	28 (39)	17 (36)
On-study deaths, *n* (%)	9 (13)	6 (13)

*Dex* dexamethasone, *ixa* ixazomib, *pom* pomalidomide, *StD* standard deviation, *TEAE* treatment-emergent adverse event.

Safety profiles are summarized in Table 3. In total, 69% vs 81% of patients had Grade ≥3 TEAEs with ixa-dex vs pom-dex, 51% vs 53% had serious TEAEs, 39% vs 36% had a TEAE leading to drug discontinuation, 44% vs 32% had a TEAE leading to dose reduction, and 13% of patients in each arm died while on study.

The most common any-grade and Grade ≥3 TEAEs with ixa-dex and pom-dex are summarized in Table 4. TEAEs occurring with a higher incidence (≥10% rate difference) with ixa-dex vs pom-dex were diarrhea (40% vs 28%), PN (29% vs 6%), insomnia (22% vs 11%), and peripheral edema (14% vs 4%). TEAEs occurring with a higher incidence (≥10% rate difference) with pom-dex vs ixa-dex were neutropenia (45% vs 3%), anemia (38% vs 18%), pruritus (11% vs 0), asthenia (17% vs 6%), and pyrexia (15% vs 4%). No Grade ≥3 TEAEs occurred at a higher incidence (≥10% rate difference) in the ixa-dex arm than the pom-dex arm. Grade ≥3 TEAEs occurring at a ≥ 10% higher rate with pom-dex vs ixa-dex were anemia (15% vs 30%) and neutropenia (3% vs 45%). The most common serious TEAE in both arms was pneumonia, reported in 9 (13%) ixa-dex vs 10 (21%) pom-dex patients. TEAEs that resulted in study drug discontinuation in ≥5% of patients were thrombocytopenia in 5 patients (7%) and PN in 4 patients (6%) in the ixa-dex arm vs pneumonia in 4 patients (9%) and neutropenia in 3 patients (6%) in the pom-dex arm. On-study deaths were not considered related to study treatment except for 1 patient in the ixa-dex arm who died due to a treatment-related AE (gastrointestinal disorder).

**Table 4:** Most common any-grade (reported in ≥10% of patients in either arm) and Grade ≥3 TEAEs (reported in ≥5% of patients in either arm), plus rates of additional TEAEs of clinical importance, reported with ixa-dex and pom-dex in the safety population.

MedDRA preferred term/higher-level term/SMQ/pooled term, *n* (%)	Ixa-dex *n* = 72		Pom-dex *n* = 47	
	Any grade	Grade ≥ 3	Any grade	Grade ≥ 3
Diarrhea	29 (40)	4 (6)	13 (28)	0
Thrombocytopenia^a^	27 (38)	23 (32)	12 (26)	9 (19)
Peripheral neuropathy^a^	21 (29)	2 (3)	3 (6)	0
Fatigue	17 (24)	1 (1)	11 (23)	2 (4)
Insomnia	16 (22)	1 (1)	5 (11)	0
Anemia	13 (18)	11 (15)	18 (38)	14 (30)
Nausea	12 (17)	1 (1)	7 (15)	0
Pneumonia	12 (17)	10 (14)	11 (23)	11 (23)
Vomiting	10 (14)	1 (1)	4 (9)	0
Peripheral edema	10 (14)	2 (3)	2 (4)	0
Arrhythmias^a^	9 (13)	4 (6)	5 (11)	4 (9)
Constipation	9 (13)	0	8 (17)	0
Rash^a^	9 (13)	1 (1)	7 (15)	0
Back pain	8 (11)	0	2 (4)	0
Bronchitis	8 (11)	2 (3)	6 (13)	0
Upper respiratory tract infection	7 (10)	0	6 (13)	0
Urinary tract infection	5 (7)	2 (3)	6 (13)	0
Asthenia	4 (6)	0	8 (17)	3 (6)
Cough	4 (6)	0	6 (13)	0
Dyspnea	4 (6)	0	5 (11)	0
Pyrexia	3 (4)	0	7 (15)	2 (4)
Neutropenia^a^	2 (3)	2 (3)	21 (45)	21 (45)
Pruritus	0	0	5 (11)	0
Other TEAEs of clinical importance				
Heart failure^a^	3 (4)	3 (4)	2 (4)	2 (4)
Renal impairment^a^	2 (3)	1 (1)	3 (6)	2 (4)
Liver impairment^a^	2 (3)	1 (1)	1 (2)	0
Hypotension^a^	1 (1)	0	3 (6)	0
Encephalopathy^a^	1 (1)	0	0	0
New primary malignancies	1 (1)		3 (6)	

*Dex* dexamethasone, *HLT* high level term, *ixa* ixazomib, *MedDRA* Medical Dictionary for Regulatory Activities, *pom* pomalidomide, *SMQ* standardized MedDRA query, *TEAE* treatment-emergent adverse event.

^a^Higher-level term, SMQ, or pooled term incorporating multiple preferred terms. Peripheral neuropathy preferred terms: neuropathy peripheral, peripheral motor neuropathy, peripheral sensory neuropathy, peripheral sensorimotor neuropathy. Thrombocytopenia preferred terms: thrombocytopenia, platelet count decreased. Rash preferred terms: acute febrile neutrophilic dermatosis, dermatitis acneiform, dermatitis allergic, drug eruption, erythema multiforme, exfoliative rash, interstitial granulomatous dermatitis, pruritus, pruritus generalized, purpura, rash, rash erythematous, rash follicular, rash generalized, rash macular, rash maculo-papular, rash maculovesicular, rash morbilliform, rash papular, rash pruritic, rash pustular, rash vesicular, red man syndrome, Stevens-Johnson syndrome, toxic epidermal necrolysis, urticaria, urticaria papular, vasculitic rash. Arrhythmias: cardiac arrhythmias SMQ (broad). Heart failure: modified cardiac failure (broad) SMQ (excluding preferred terms of edema, edema peripheral, and peripheral swelling). Neutropenia preferred terms: neutropenia, neutrophil count decreased. Renal impairment: acute renal failure SMQ (broad). Liver impairment: cholestasis and jaundice of hepatic origin SMQ (broad), hepatic failure, fibrosis, cirrhosis and other liver damage-related conditions SMQ (broad), liver-related investigations signs and symptoms SMQ (broad). Hypotension: modified vascular hypotensive disorders HLT (excluding preferred terms of systemic inflammatory response syndrome and vasoplegia syndrome), vascular test HLT (including only preferred terms related to blood pressure decreased). Encephalopathy: non-infectious encephalopathy/delirium SMQ (narrow). Myocardial infarction: myocardial infarction SMQ (broad).

### Patient-reported outcomes and healthcare resource utilization

**Fig. 4:**
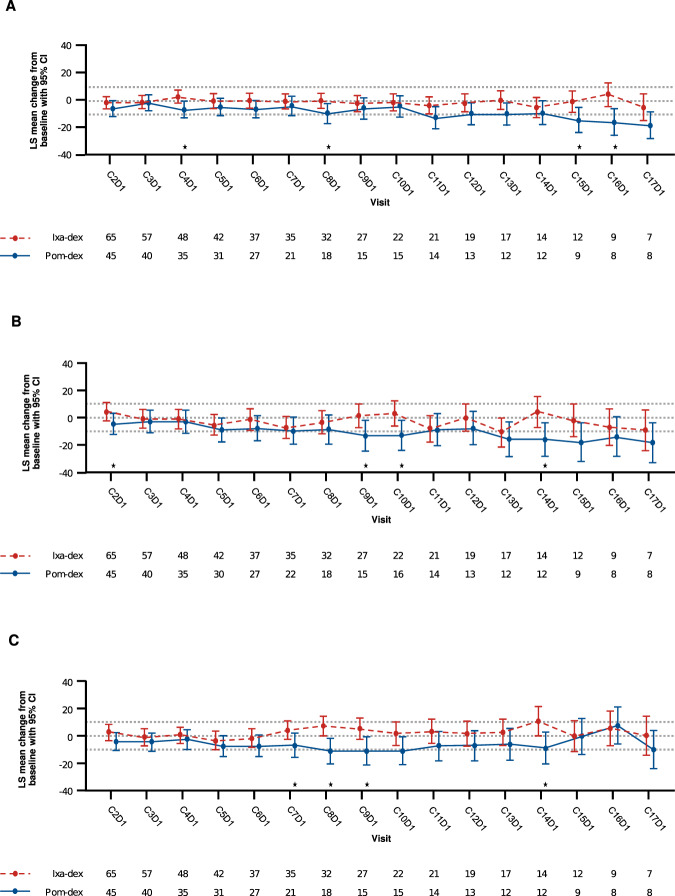
Mean scores over time for EORTC QLQ-C30 domains with ixa-dex vs pom-dex. Significant differences between arms were seen at multiple time points: (**A**) cognitive functioning, (**B**) insomnia, and (**C**) diarrhea in patients on the ixa-dex and pom-dex arms. C Cycle, D1 Day 1, dex dexamethasone, CI confidence interval, ixa ixazomib, LS least squares, pom pomalidomide. * Indicates *P* ≤ 0.05.

HRQoL, as assessed by mean domain scores on the patient-reported EORTC QLQ-C30 and MY-20 instruments, was generally similar at baseline between the 2 arms and was maintained over time. On the EORTC QLQ-C30 instrument, functional subscale scores were generally high at baseline, indicating good HRQoL, whereas symptom scale scores for fatigue, pain, and nausea/vomiting were high or very high (Supplementary Table 4). Significant differences between treatment arms were seen at multiple time points for the cognitive functioning (Cycles 4, 8, 15, and 16), insomnia (Cycles 2, 9, 10, and 14), and diarrhea (Cycles 7, 8, 9, and 14) domains. For these 3 scales, mean changes from baseline in the ixa-dex arm were somewhat higher compared with those in the pom-dex arm, indicating better cognitive functioning but worse insomnia and diarrhea symptoms (Fig. 4). Mean baseline scores for the EORTC QLQ-MY20 subscales were high and similar between arms, and remained stable and similar over time (Supplementary Fig. 4).

HRU during treatment is summarized in Supplementary Table 5. Mean rates of hospitalizations and emergency room stays were similar in the ixa-dex and pom-dex arms, whereas the rate of outpatient visits per patient-year exposure was higher in the pom-dex arm.

## Discussion

This Phase 2 study demonstrated that PFS was comparable with ixa-dex and pom-dex in this population of heavily pretreated, carfilzomib and/or bortezomib-exposed and/or intolerant, lenalidomide-refractory patients (with medians of 7.1 and 4.8 months, respectively, and a HR of 0.847); there was no statistically significant difference between arms. These PFS data for ixa-dex appear generally consistent with median event-free survival findings of 7.8–11.5 months reported across dosing regimens in a previous Phase 2 study [35, 36], allowing for the higher rate of lenalidomide-refractory patients in the present study. Median PFS with pom-dex appeared similar or slightly higher than the 4.0- and 4.6-month medians seen in previous Phase 3/3b studies of pom-dex in refractory populations [39, 40], but slightly lower than the 6.9 months reported in the APOLLO Phase 3 study in less heavily pretreated patients [23].

Subgroup analyses of PFS showed numerically lower HRs in patients with ≥3 prior lines of therapy (HR 0.686) and in patients with creatinine clearance <60 mL/min (HR 0.467) compared to the ITT analysis (HR 0.847). The PFS HR appeared comparable in patients aged <75 (HR 0.843) and ≥75 (HR 0.890) years suggesting that, although there was an imbalance in the proportions of elderly patients between arms, this was unlikely to have materially impacted PFS findings in the ITT population.

Secondary efficacy endpoints appeared comparable between treatment arms, including ORR (38% vs 41%), TTR (median 2.0 vs 1.1 months), and DOR (median 14.8 vs 14.3 months). The TTP HR (0.830) was similar to that of PFS.

OS data were not mature at data cutoff, with only 36% of patients having died, and the study was not powered for OS comparisons. Nevertheless, preliminary outcomes exceeded expectations for this patient population in both arms, as previous studies report medians of 4–5 months and ~12–15 months for PFS and OS, respectively [39–41]. Differential OS findings were seen in patients aged <75 and ≥75 years. Given the advanced age of some patients, the overall OS findings may have been impacted and rendered inconclusive by the age imbalance between arms, including a higher rate of early censoring in the ixa-dex arm. The OS findings may also be partly explained by the differences in subsequent therapies received in each arm. More patients in the pom-dex arm received subsequent therapies, which may have been because they progressed sooner on study treatment, and were younger, and thus more likely to be able to receive subsequent therapies. Patients on the pom-dex arm also received subsequent daratumumab (28% vs 15%) and PIs (carfilzomib 28% vs 7%, bortezomib 13% vs 1%) more frequently than those on the ixa-dex arm who, by contrast, received pomalidomide (28% vs 4%) more frequently. Greater use of these alternative active regimens following treatment in this study may have contributed to the OS seen in the pom-dex arm, with a high proportion of patients censored at >12 months on the Kaplan–Meier analysis.

The data showed ixa-dex and pom-dex to be similarly tolerable. Notably, ixazomib was sufficiently well tolerated at the starting dose of 4 mg (based on the dose used in TOURMALINE-MM1 [26]) to enable 64% of patients to escalate to the 5.5 mg dose. This dose escalation of ixazomib was based on the previous Phase 2 study of ixa-dex, in which greater efficacy was seen with an ixazomib dose of 5.5 mg vs 4 mg (ORR 54% vs 31%), albeit with higher rates of TEAEs [35, 36]. In the current study, median duration of treatment with ixazomib 5.5 mg was 1.8 months (equating to ~2 cycles), suggesting that this was a challenging dose for patients and that subsequent dose reductions were required for continued treatment with ixazomib. Nonetheless, rates of discontinuations due to TEAEs were similar between the 2 arms. Thus, the oral PI-based doublet of ixa-dex appears tolerable in this treatment setting. While other approved PIs (bortezomib and carfilzomib) have demonstrated efficacy benefits in RRMM patients, these PIs require parenteral administration, a procedure with greater impact on these RRMM patients, who are less tolerant of visits to the hospital or clinic as their disease progresses [24].

There were no consistent differences between ixa-dex and pom-dex in terms of overall safety profile. Differential rates of specific TEAEs associated more commonly with ixazomib or pomalidomide were seen, such as diarrhea and thrombocytopenia with ixazomib, and anemia and pneumonia with pomalidomide [39, 40, 42]. Of note, the overall rate of PN, a TEAE of clinical importance with bortezomib-based therapy [43], was higher with ixa-dex vs pom-dex (29% vs 6%); however, this rate is consistent with that reported for ixazomib (4 mg) in combination with lenalidomide-dexamethasone in the Phase 3 TOURMALINE-MM1 trial (27%) [26], despite the higher ixazomib dose used in some patients in the current study. Overall rates of serious TEAEs in both arms were also consistent with rates of grade 3/4 treatment-related TEAEs in previous studies of ixa-dex [35, 36] and of serious TEAEs in previous studies of ixa-dex plus lenalidomide [26] and pom-dex [39, 40] in RRMM.

Rates of cardiovascular and renal toxicities, which can be associated with carfilzomib-based treatment [44], were notably low with ixa-dex suggesting that this PI-based doublet regimen may be preferred for patients at risk of those toxicities.

The tolerable safety profiles of ixa-dex and pom-dex were reflected in the similar patient-reported QoL and HRU data between arms. HRQoL scores were maintained from study entry in both arms and were generally similar throughout the study, indicating no relative adverse impact on HRQoL of one regimen vs the other.

This study had several limitations. The patient population was generally older than anticipated, and thus the age cutoff for stratification (65 years) was too low to balance the treatment arms. Consequently, there was an imbalance of 36% vs 18% in the proportions of patients aged ≥75 years in the ixa-dex vs pom-dex arms. Data on cytogenetic abnormalities were not collected routinely in this study, and so the impact of high-risk cytogenetics on outcomes could not be evaluated. However, additional stratification according to cytogenetic risk would not have been feasible due to the limited sample size, as stratification group sizes would have been too small due to the use of multiple stratification factors. Finally, as noted, OS data were not mature at data cutoff, and may have been impacted by imbalances in subsequent therapies, confounding interpretation of the findings.

In conclusion, these results suggest ixa-dex can be effective in heavily pretreated, lenalidomide-refractory, PI-exposed RRMM patients, a population with considerable unmet medical needs. Ixa-dex was tolerable, with a HRQoL comparable with pom-dex in this population. RRMM is a heterogeneous disease, and no single treatment benefits all patients. Ixa-dex is an appropriate option for patients who cannot tolerate triplet combinations, or who are not able or prefer not to receive parenteral treatment administration, or who require a non-immunomodulatory drug-based treatment approach.

## Supplementary information

Supplementary Information

## Data Availability

The datasets, including the redacted study protocol, redacted statistical analysis plan, and individual participants’ data supporting the results reported in this article will be available 3 months from initial request to researchers who provide a methodologically sound proposal. The data will be provided after its de-identification, in compliance with applicable privacy laws, data protection, and requirements for consent and anonymization.
