# Supplementary material for: Oral ixazomib-dexamethasone vs oral pomalidomide-dexamethasone for lenalidomide-refractory, proteasome inhibitor-exposed multiple myeloma: a randomized Phase﻿ 2 trial
Source: Blood Cancer J. 2022 Jan 24;12(1):9. doi: 10.1038/s41408-021-00593-2 (PMC8786921; doi:10.1038/s41408-021-00593-2)
Supplement: Supplementary file 1 — Supplementary Information [file 41408_2021_593_MOESM1_ESM.docx]

**SUPPLEMENTARY INFORMATION**

SUPPLEMENTAL TABLES

Supplemental Table 1 Inclusion and exclusion criteria

| **Inclusion criteria** | **Exclusion criteria** |
| --- | --- |
| 1. Male or female patients aged 18 years or older.  2. Had a confirmed diagnosis of MM requiring therapy according to IMWG criteria.  3. Had ECOG performance status of 0 to 2.  4. Had a relapse or PD after having received 2 or more prior lines of systemic therapy. Note: A line of therapy was defined as 1 or more cycles of a planned treatment program; this may have consisted of 1 or more planned cycles of single-agent therapy or combination therapy, as well as a sequence of treatments administered in a planned manner. For example, a planned treatment approach of induction therapy followed by ASCT, followed by maintenance was considered 1 line of therapy. Typically, each line of therapy is separated by PD.  5. Was refractory to lenalidomide, defined as having received at least 2 consecutive cycles of lenalidomide as a single agent or within a lenalidomide-containing regimen and having had PD during treatment with or within 60 days after the last dose of lenalidomide. The starting dose of lenalidomide should have been 25 mg (or as low as 10 mg in the case of renal function impairment or other safety concern), and the final dose should have been a minimum of 10 mg.  6. Received at least 2 consecutive cycles of a bortezomib- or carfilzomib-containing regimen, and either:  – Achieved at least a PR and did not have PD during treatment with or within 60 days after the last dose of bortezomib or carfilzomib, OR  – Had bortezomib and/or carfilzomib intolerance (defined as discontinuation because of drug-related AEs before completion of the planned treatment course) without PD before the start of the next regimen.  7. Had measurable disease defined by:  – Serum M-protein ≥1 g/dL (≥10 g/L), OR  – Urine M-protein ≥200 mg/24 hours and must have had documented MM isotype by immunofixation (central laboratory).  8. Met all the following clinical laboratory criteria:  – ANC ≥1000/mm^3^ and platelet count ≥75,000/mm^3^, without growth factor or transfusion support.  – Total bilirubin ≤1.5 times the ULN.  – ALT and AST ≤3 times the ULN.  – Calculated CrCl ≥30 mL/min.  9. Female patients who:  – Were postmenopausal for at least 1 year before the screening visit, OR  – Were surgically sterile, OR  – If they were of childbearing potential, agreed to practice 1 highly effective method of contraception and 1 additional effective (barrier) method at the same time, for 4 weeks before signing the ICF through 90 days after the last dose of study therapy, OR  – Agreed to practice true abstinence, when this was in line with the preferred and usual lifestyle of the patient. (Periodic abstinence [e.g., calendar, ovulation, symptothermal, postovulation methods], withdrawal, spermicides only, and lactational amenorrhea were not acceptable methods of contraception. Female and male condoms were not to be used together), AND  – In women of childbearing potential (if randomized to the pom+dex arm), agreed to have 2 negative pregnancy tests before initiating therapy, with 1 or both being a serum test (the first test should be performed within 10 to 14 days before; the second, within 24 hours before); then were to have a negative pregnancy test weekly during the first month and monthly thereafter in women with regular menstrual cycles or every 2 weeks thereafter in women with irregular menstrual cycles; and were to have a negative pregnancy test 4 weeks after the last dose of study therapy.  10. Male patients, even if surgically sterilized (i.e., status postvasectomy), who:  – Agreed to practice effective barrier contraception during the entire study treatment period and through 90 days after the last dose of study therapy, OR  – Agreed to practice true abstinence, when this was in line with the preferred and usual lifestyle of the patient. (Periodic abstinence [e.g., calendar, ovulation, symptothermal, postovulation methods], withdrawal, spermicides only, and lactational amenorrhea were not acceptable methods of contraception. Female and male condoms were not to be used together.), AND  – Agreed not to donate semen or sperm during treatment and for 90 days after the last dose of study therapy.  11. Gave voluntary written consent before performance of any study-related procedure not part of standard medical care, with the understanding that consent could have been withdrawn by the patient at any time without prejudice to future medical care.  12. Had suitable venous access for the study-required blood sampling, including PK sampling.  13. Was willing and able to adhere to the study visit schedule and other protocol requirements including blood sampling and bone marrow aspiration.  14. Recovered (i.e., Grade ≤1 non-hematological toxicity) from the reversible effects of prior anticancer therapy.  15. Was willing and able to adhere to pomalidomide-related risk mitigation activities if randomized to the pom+dex arm (e.g., risk evaluation and mitigation strategies, pregnancy prevention programs). | 1. Prior ixazomib or pomalidomide treatment and was a participant in a previous ixazomib clinical study.  2. Prior allogenic bone marrow transplantation in any prior line of therapy or prior ASCT in the last prior line of therapy—unless the ASCT was performed a year or more before disease progression.  3. Female patients who were lactating and breastfeeding or had a positive serum pregnancy test during the screening period.  4. Any serious medical or psychiatric illness that could have, in the investigator’s opinion, potentially interfered with the completion of treatment according to this protocol, such as life-threatening illness unrelated to cancer.  5. Diagnosed with or treated for another malignancy within 2 years before randomization, or previously diagnosed with another malignancy and had any evidence of residual, persistent, or recurrent disease. Patients with nonmelanoma skin cancer or carcinoma *in situ* of any type were not excluded if they had undergone complete resection.  6. Diagnosis of smoldering MM, Waldenström’s macroglobulinemia, POEMS syndrome, plasma cell leukemia, primary amyloidosis, myelodysplastic syndrome, or myeloproliferative syndrome.  7. Known allergy to any of the study medications or their analogs, or excipients in the various formulations.  8. Peripheral neuropathy Grade 1 with pain or Grade 2 or higher peripheral neuropathy of any cause on clinical examination during the screening period.  9. Treatment with any investigational products or with chimeric or fully human monoclonal antibodies within 30 days before randomization, systemic anticancer therapy or radiotherapy within 14 days before randomization (Note: “spot” radiation for areas of pain was permitted), and major surgery within 14 days before randomization.  10. Known gastrointestinal disease or gastrointestinal procedure that could interfere with the oral absorption or tolerance of study therapy, including difficulty swallowing.  11. Serious infection requiring parenteral antibiotic therapy or any other serious infection within 14 days before randomization.  12. Central nervous system involvement with MM (by clinical symptoms and signs).  13. Ongoing or active systemic infection, known HIV-RNA positive, known hepatitis B surface antigen seropositive, or known hepatitis C virus-RNA positive.  Note: Patients with positive hepatitis B core antibody could be enrolled but had to be hepatitis B virus-DNA negative. Patients with positive hepatitis C antibody could be enrolled but had to be hepatitis C virus-RNA negative.  14. Systemic treatment with strong cytochrome P-450 3A inducers (rifampin, rifapentine, rifabutin, carbamazepine, phenytoin, phenobarbital) or use of St. John’s wort within 14 days before randomization.  15. Admission or evidence of illicit drug use, drug abuse, or alcohol abuse.  16. History of severe cutaneous reactions, including hypersensitivity reactions, such as Stevens-Johnson syndrome, Toxic Epidermal Necrolysis, and Drug Reaction with Eosinophilia and Systemic Symptoms, in the context of treatment with lenalidomide or thalidomide. |

*AE* adverse event, *ALT* alanine aminotransferase, *ANC* absolute neutrophil count, *ASCT* autologous stem cell transplant, *AST* aspartate aminotransferase, *CrCl* creatinine clearance, d*ex* dexamethasone, *ECOG* Eastern Cooperative Oncology Group, *ICF* informed consent form, *IMWG* International Myeloma Working Group, *MM* multiple myeloma, *PD* progressive disease, *PK* pharmacokinetic, *POEMS* polyneuropathy, organomegaly, endocrinopathy, monoclonal gammopathy, and skin changes, *pom* pomalidomide, *PR* partial response, *ULN* upper limit of normal.

Supplemental Table 2 Prior lines of therapy received before ixa-dex or
pom-dex in the ITT population.

| Patients with prior lines of therapy containing the listed agents, *n* (%) | Ixa-dex  *N* = 73 | Pom-dex  *N* = 49 |
| --- | --- | --- |
| Corticosteroids | 72 (99) | 48 (98) |
| Dexamethasone | 72 (99) | 46 (94) |
| Prednisone | 27 (37) | 14 (29) |
| PIs | 73 (100) | 49 (100) |
| Bortezomib | 73 (100) | 49 (100) |
| Carfilzomib | 7 (10) | 1 (2) |
| Alkylating agents | 65 (89) | 44 (90) |
| Bendamustine | 4 (5) | 2 (4) |
| Cyclophosphamide | 48 (66) | 33 (67) |
| Melphalan | 38 (52) | 29 (59) |
| Immunomodulatory drugs | 73 (100) | 49 (100) |
| Lenalidomide | 73 (100) | 49 (100) |
| Thalidomide | 23 (32) | 10 (20) |
| Platinum compounds – Cisplatin | 1 (1) | 0 |
| Monoclonal antibody – Daratumumab | 14 (19) | 7 (14) |
| Best response to prior antineoplastic therapy, *n* (%)^a^ |  |  |
| CR | 14 (19) | 14 (29) |
| VGPR | 33 (45) | 20 (41) |
| PR | 26 (36) | 15 (31) |
| Prior radiation | 17 (23) | 11 (22) |
| Prior transplant | 32 (44) | 28 (57) |

*CR* complete response, d*ex* dexamethasone, *ITT* intent-to-treat, *ixa* ixazomib, *PD* progressive disease, *PI* proteasome inhibitor, *pom* pomalidomide, *PR* partial response, *VGPR* very good partial response.

^a^Percentages are based on the number of patients with prior antineoplastic therapy/prior radiation in each column.

Supplemental Table 3 Subsequent antineoplastic therapies received by ≥3% of patients in the safety population of either treatment arm.

| Patients with subsequent therapy, *n* (%) | Ixa-dex  *N* = 72 | Pom-dex  *N* = 47 |
| --- | --- | --- |
| Patients with ≥1 subsequent anti-MM therapy | 30 (42) | 23 (49) |
| Corticosteroids | 20 (28) | 16 (34) |
| Dexamethasone | 19 (26) | 16 (34) |
| Immunomodulatory drugs | 23 (32) | 4 (9) |
| Pomalidomide | 20 (28) | 2 (4) |
| Lenalidomide | 2 (3) | 1 (2) |
| mABs specific to MM therapy | 14 (19) | 13 (28) |
| Daratumumab | 11 (15) | 13 (28) |
| Elotuzumab | 2 (3) | 0 |
| PIs | 7 (10) | 17 (36) |
| Carfilzomib | 5 (7) | 13 (28) |
| Bortezomib | 1 (1) | 6 (13) |
| Ixazomib | 1 (1) | 2 (4) |
| Alkylating agents | 7 (10) | 6 (13) |
| Cyclophosphamide | 6 (8) | 5 (11) |
| Other | 1 (1) | 2 (4) |

*Dex* dexamethasone, *ixa* ixazomib, *mABs* monoclonal antibodies, *MM* multiple myeloma, *PI* proteasome inhibitor, *pom* pomalidomide

Supplemental Table 4 EORTC QLQ-C30 domain scores at baseline in the ITT PRO populations of the ixa-dex
and pom-dex arms.

| Baseline EORTC QLQ-C30 domain score:  mean [95% CI], median (range) | Ixa-dex  *N* = 70 | Pom-dex  *N* = 45 |
| --- | --- | --- |
| Global health status | 60.8 [55.8–65.9], 58.3 (8–100) | 57.0 [50.8–63.3], 58.3 (17–100) |
| *QOL functional scales* |  |  |
| Physical functioning | 67.9 [62.6–73.1], 70.0 (20–100) | 67.0 [60.4–73.6], 73.3 (0–100) |
| Role functioning | 67.9 [60.8–74.9], 66.7 (0–100) | 67.4 (58.9–75.9), 66.7 (0–100) |
| Emotional functioning | 83.1 [77.9–88.3], 91.7 (0–100) | 74.9 [68.5–81.2], 75.0 (17–100) |
| Cognitive functioning | 84.0 [79.1–89.0], 100.0 (33–100) | 79.6 [73.5–85.8], 83.3 (33–100) |
| Social functioning | 75.7 [69.3–82.2], 83.3 (0–100) | 72.2 [63.2–81.2], 83.3 (33–100) |
| *Symptom scales/items* |  |  |
| Fatigue | 61.0 [55.5–66.5], 66.7 (0–100) | 60.0 [53.1–66.9], 66.7 (0–100) |
| Nausea and vomiting | 94.8 [92.0–97.5], 100.0 (50–100) | 96.7 [94.1–99.2], 100.0 (67–100) |
| Pain | 65.2 [59.3–71.1], 66.7 (0–100) | 61.1 [51.9–70.4], 66.7 (0–100) |
| Dyspnea | 19.0 [12.9–25.2], 0.0 (0–100) | 25.2 [16.6–33.8], 33.3 (0–100) |
| Insomnia | 29.5 [21.9–37.1], 33.3 (0–100) | 32.6 [23.7–41.5], 33.3 (0–100) |
| Appetite loss | 14.3 [8.9–19.6], 0.0 (0–100) | 22.2 [14.0–30.5], 0.0 (0–100) |
| Constipation | 11.4 [6.6–16.3], 0.0 (0–67) | 13.3 [6.1–20.5], 0.0 (0–100) |
| Diarrhea | 16.7 [11.1–22.2], 0.0 (0–67) | 17.8 [10.8–24.7] 0.0 (0–100) |
| Financial difficulties | 17.1 [11.0–23.3], 0.0 (0–100) | 22.2 [14.0–30.5], 0.0 (0–100) |

*CI* confidence interval, *dex* dexamethasone, *EORTC QLQ-C30* European Organization for Research and Treatment of Cancer Quality of Life Questionnaire-Core-30, *ITT* intent-to-treat, *ixa* ixazomib, *QOL* quality of life, *pom* pomalidomide, *PRO* patient-reported outcomes.

**Supplemental Table 5 HRU during treatment with ixa-dex and pom-dex in the ITT population.**

| Healthcare resource | Ixa-dex  *N* = 73  TPY = 73^a^ | Pom-dex  *N* = 49  TPY = 53^a^ |
| --- | --- | --- |
| *Hospitalizations^b^* |  |  |
| Number of patients with ≥1 hospitalization, *n* (%) | 23 (32) | 16 (33) |
| Number of hospitalizations, *n* | 45 | 38 |
| Mean number of hospitalizations per patient, *n* (StD) | 2.0 (1.97) | 2.4 (1.50) |
| Rate of hospitalizations per patient-year [95% CI]^c^ | 0.6146 [0.4350–0.7941] | 0.7123 [0.4858–0.9388] |
| Median length of time spent in hospital for patients with ≥1 hospitalization, days (range) | 12.0 (2–66) | 13.0 (4–111) |
| *ER stays* |  |  |
| Number of patients with ≥1 ER stay, *n* (%) | 11 (15) | 9 (18) |
| Number of ER stays, *n* | 14 | 13 |
| Mean number of ER stays per patient, *n* (StD) | 1.3 (0.65) | 1.4 (0.73) |
| Rate of ER stays per patient-year [95% CI]^c^ | 0.1912 [0.0910–0.2913] | 0.2437 [0.1112–0.3762] |
| *Outpatient visits* |  |  |
| Number of patients with ≥1 outpatient visit, *n* (%) | 32 (44) | 29 (59) |
| Number of outpatient visits, *n* | 138 | 227 |
| Mean number of outpatient visits per patient, *n* (StD) | 4.3 (5.98) | 7.8 (11.28) |
| Rate of outpatient visits per patient-year [95% CI]^c^ | 1.8846 [1.5702–2.1991] | 4.2552 [3.7016–4.8087] |

*CI* confidence interval, *dex* dexamethasone, *ER* emergency room, *HRU* healthcare resource utilization, *ITT* intent-to-treat, *ixa* ixazomib, *PFS* progression-free survival, *pom* pomalidomide, *PRO* patient-report outcome, *StD* standard deviation, *TPY* total patient years.

^a^Total patient-year is the sum of patient-year of all subjects in the treatment group, where patient-year was defined as [(the earlier of the last PFS follow-up date, cutoff date or date of death) — first dose date + 1] / 365.25.
^b^A hospitalization was defined as at least 1 overnight stay in an intensive care unit and/or non-intensive care unit (acute care unit, palliative care unit, or hospice). A single hospitalization could include staying in an intensive care unit and/or non-intensive care unit.

^c^Rate was calculated as the total number of events divided by the total number of patient-years in each group. The lower limit of 95% CI is truncated at 0 when it is negative.

SUPPLEMENTAL FIGURES

Supplemental Figure 1 Kaplan–Meier analysis by investigator assessment based on age of the ITT population: A) PFS in patients aged <75 years; B) PFS in patients aged ≥75 years; C) OS in patients aged <75 years; and D) OS in patients aged ≥75 years.

**Probability of PFS**

0

**Time from randomization, months**

1.0

6

0.8

0.0

0.6

0.4

0.2

3

9

12

15

18

21

24

27

**Patients at risk, *n***

40

14

25

11

9

6

4

1

1

0

Pom-dex

30

47

20

29

17

11

6

2

2

0

0

Ixa-dex

0

0

Pom-dex

Censored

Ixa-dex

Censored

Log-Rank test *P* value: 0.536

HR (95% CI): 0.843 (0.491–1.448)

Median:

Events, *n*:

Ixa-dex: 6.5

Ixa-dex: 31

Pom-dex: 4.8

Pom-dex: 27

**B)**

**Probability of PFS**

1.0

**Time from randomization, months**

0.0

0.8

0.6

0.4

0.2

**Patients at risk, *n***

0

9

6

3

3

8

9

3

12

3

15

3

18

0

Pom-dex

26

11

16

5

4

2

0

Ixa-dex

Pom-dex

Censored

Ixa-dex

Censored

Log-Rank test *P* value: 0.828

HR (95% CI): 0.890 (0.309–2.562)

Median:

Events, *n*:

Ixa-dex: 8.0

Ixa-dex: 15

Pom-dex: 4.7

Pom-dex: 7

21

24

27

30

0

0

0

0

0

0

0

0

**C)**

**Probability of OS**

0

**Time from randomization, months**

1.0

6

0.0

0.8

0.6

0.4

0.2

3

9

12

15

18

21

24

30

**Patients at risk, *n***

40

31

35

29

22

18

13

8

5

0

Pom-dex

27

1

47

34

39

29

21

17

11

4

1

0

Ixa-dex

0

Pom-dex

Censored

Ixa-dex

Censored

Log-Rank test *P* value: 0.218

HR (95% CI): 1.595 (0.754–3.375)

Median:

Events, *n*:

Ixa-dex: 18.8

Ixa-dex: 19

Pom-dex: NE

Pom-dex: 11

**D)**

**Probability of OS**

0

1.0

0.8

0.6

0.4

0.2

0.0

6

3

9

12

15

18

21

24

27

**Time from randomization, months**

**Patients at risk, *n***

9

7

Pom-dex

Censored

Log-Rank test *P* value: 0.838

HR (95% CI): 0.873 (0.237–3.214)

Median:

Events, *n*:

Ixa-dex: NE

Ixa-dex: 10

Pom-dex: NE

Pom-dex: 4

Ixa-dex

Censored

30

8

6

26

20

24

12

10

7

4

3

1

0

Ixa-dex

0

4

4

2

1

0

0

Pom-dex

0

*CI* confidence interval, *dex* dexamethasone, *HR* hazard ratio, *ITT* intent-to-treat, *ixa* ixazomib, *NE* not estimable, *OS* overall survival, *PFS* progression-free survival, *pom* pomalidomide.

Supplemental Figure 2 Kaplan-Meier analysis of DOR by investigator assessment in the response-evaluable population.

**Probability of DOR**

1.0

0.0

0.8

0.6

0.4

0.2

**Time from randomization, months**

**Patients at risk, *n***

0

6

3

9

12

15

18

21

24

20

14

Pom-dex

Censored

Median:

Events, *n*:

Ixa-dex: 14.8

Ixa-dex: 10

Pom-dex: 14.3

Pom-dex: 10

Ixa-dex

Censored

16

9

6

4

1

1

0

Pom-dex

28

18

23

13

5

3

2

1

0

Ixa-dex

*Dex* dexamethasone, *DOR* duration of response, *ixa* ixazomib, *pom* pomalidomide.

**Supplemental Figure 3 Forest plot of OS in prespecified subgroups based on patient and disease characteristics in the ITT population.**

**
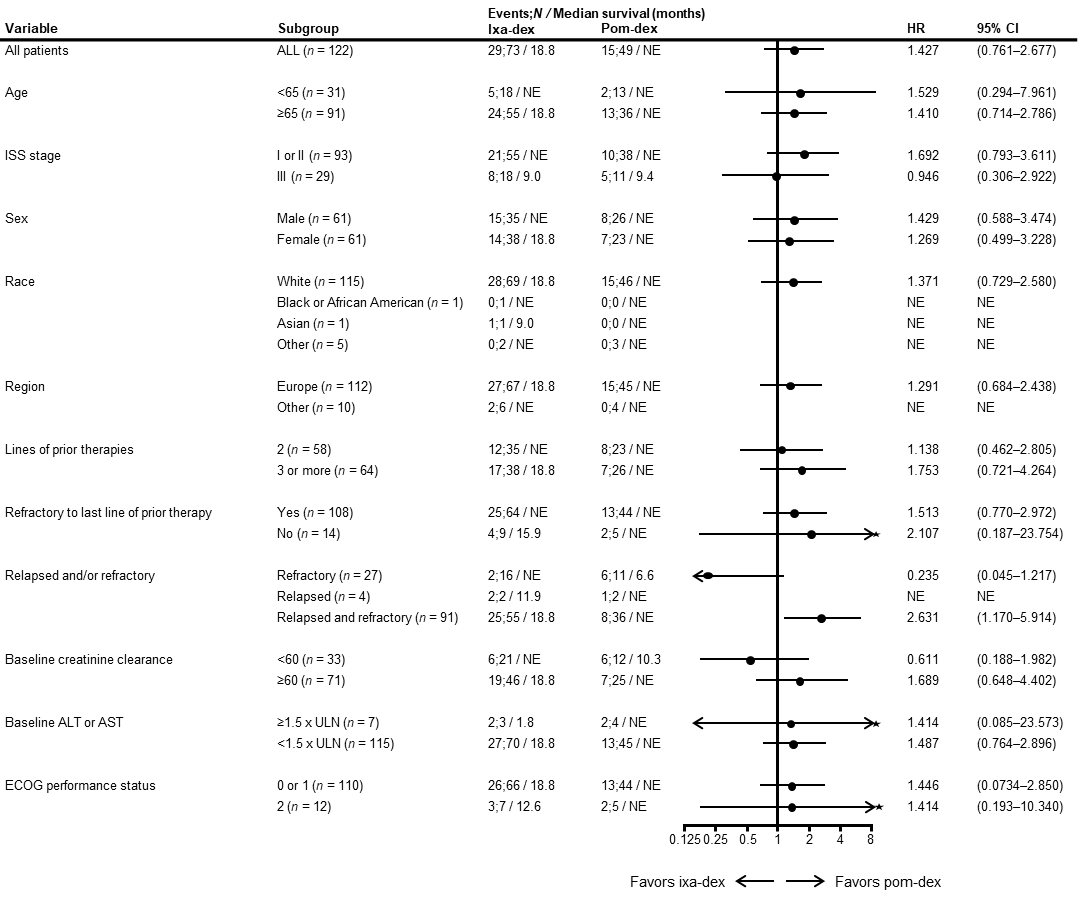
**

*ALT* alanine aminotransferase, *AST* aspartate aminotransferase, *CI* confidence interval, *dex* dexamethasone, *ECOG* Eastern Cooperative Oncology Group, *HR* hazard ratio, *ISS* International Staging System, *ITT* intent-to-treat, *ixa* ixazomib, *NE* not estimable, *OS* overall survival, *pom* pomalidomide, *ULN* upper limit of normal.

Supplemental Figure 4 EORTC QLQ-MY20 subscale scores at baseline, on Day 1 of each cycle of treatment, and at PFS follow-up visits: A) disease symptoms; B) side effects of treatment; C) body image; and D) future perspective.
A)

Pom-dex

44

45

35

40

27

30

18

21

16

15

13

14

12

12

8

9

7

7

5

7

3

4

2

3

2

2

1

1

1

Ixa-dex

70

65

57

48

42

37

34

32

27

22

21

19

17

14

12

9

7

5

5

4

2

2

2

2

2

0

0

0

3

3

**Mean score with 95% CI**

100

80

60

40

0

20

BL

C2D1

C3D1

C4D1

C5D1

C6D1

C7D1

C8D1

C9D1

C10D1

C11D1

C12D1

C13D1

C14D1

C15D1

C16D1

C17D1

C18D1

C19D1

C20D1

C21D1

C22D1

C23D1

C24D1

C25D1

C26D1

C27D1

C28D1

PFS01

PFS02

PFS03

**Visit**

1

1

1

**B)**

**Mean score with 95% CI**

100

80

60

40

0

20

**Visit**

C2D1

C3D1

C4D1

C5D1

C6D1

C7D1

C8D1

C9D1

C10D1

C11D1

C12D1

C13D1

C14D1

C15D1

C16D1

C17D1

C18D1

C19D1

C20D1

C21D1

C22D1

C23D1

C24D1

C25D1

C26D1

C27D1

C28D1

PFS01

PFS02

PFS03

BL

70

65

57

48

42

37

34

32

27

22

21

19

17

14

12

9

7

5

5

4

2

2

2

2

2

0

0

0

3

3

1

44

45

35

40

27

30

18

21

16

15

13

14

12

12

8

9

7

7

5

7

3

4

2

3

2

2

1

1

1

1

1

Ixa-dex

Pom-dex

**C)**

**Mean score with 95% CI**

100

80

60

40

0

20

C2D1

C3D1

C4D1

**Visit**

43

45

35

39

27

30

18

21

15

15

13

14

12

12

8

9

7

7

5

7

3

4

2

3

2

2

1

0

1

1

C5D1

C6D1

C7D1

C8D1

C9D1

C10D1

C11D1

C12D1

C13D1

C14D1

C15D1

C16D1

C17D1

C18D1

C19D1

C20D1

C21D1

C22D1

C23D1

C24D1

C25D1

C26D1

C27D1

C28D1

PFS01

PFS02

PFS03

BL

1

70

64

56

48

42

37

35

32

27

22

21

19

17

14

12

9

7

5

5

4

2

2

2

2

2

0

0

0

3

3

1

Pom-dex

Ixa-dex

**Mean score with 95% CI**

100

80

60

40

20

0

C2D1

C3D1

C4D1

**Visit**

44

45

35

40

27

30

18

22

15

15

13

14

12

12

8

9

7

7

5

7

3

4

2

3

2

2

1

0

1

C5D1

C6D1

C7D1

C8D1

C9D1

C10D1

C11D1

C12D1

C13D1

C14D1

C15D1

C16D1

C17D1

C18D1

C19D1

C20D1

C21D1

C22D1

C23D1

C24D1

C25D1

C26D1

C27D1

C28D1

PFS01

PFS02

PFS03

BL

Pom-dex

1

1

Ixa-dex

70

64

57

48

42

37

35

32

27

22

21

19

17

14

12

9

7

5

5

4

2

2

2

2

2

0

0

0

3

3

1

**D)**

*BL*, baseline, *C* Cycle, *CI* confidence interval, *D* Day, *dex* dexamethasone*, EORTC* European Organization for Research and Treatment of Cancer, *ixa* ixazomib, *pom* pomalidomide, *PFS* progression-free survival*, QLQ-MY20* quality of life questionnaire, disease-specific multiple myeloma module.
